# Impact of parental exercise on epigenetic modifications inherited by offspring: A systematic review

**DOI:** 10.14814/phy2.14287

**Published:** 2019-11-23

**Authors:** Jessie E. Axsom, Joseph R. Libonati

**Affiliations:** ^1^ Department of Nursing Science University of Pennsylvania Philadelphia PA USA

**Keywords:** disease prevention, DNA methylation, epigenetics, epigenome, exercise, intergenerational, metabolism, sperm

## Abstract

Performing regular exercise is associated with numerous health benefits including a reduction in all‐cause mortality. The mechanisms associated with exercise‐induced health improvements are wide ranging and benefit virtually every organ system in the body. Of significance, recent evidence has suggested that some of these protective benefits may also be passed to offspring through multiple generations via alterations in gamete presentation, changes to the in‐utero and offspring rearing environments, and epigenetic modifications. The purpose of this review was to systematically examine the current literature for evidence of exercise‐induced epigenetic modifications in offspring. A systematic search yielded four papers that met inclusion criteria. Parental exercise interventions were associated with differential DNA methylation patterns in offspring. These shifts in methylation patterns were consistent with concurrent changes in offspring mRNA levels, protein expression, and functional measures. Many of the observed changes were related to metabolic pathways. Hence, the evidence suggests that exercise‐induced epigenetic changes can be observed in offspring and may play a pivotal role among the multifactorial intergenerational‐health impact of exercise.

## Abstract 2

A proposed mechanism for the wide‐ranging health benefits of exercise is epigenetic changes and there is potential for epigenetic changes to be passed on to offspring through intergenerational inheritance.

## Graphical abstract

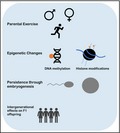

## 1 INTRODUCTION

Exercise is a powerful form of preventative medicine, offering protection from a wide‐range of chronic diseases. For example, Nocon et al. (2008) found increased levels of physical activity can reduce cardiovascular‐associated mortality by 35% and all‐cause mortality by 33% (Nocon et al., 2008) in both cardiovascular risk‐factor adjusted and unadjusted populations (Nocon et al., 2008). Physical activity prevents hypertension, hypercholesteremia, type‐II diabetes, metabolic syndrome, and alters inflammatory biomarkers—all of which are associated with the development of cardiovascular disease (Lee et al., 2012; Liu et al., 2018). The physiologic benefits of regular physical activity extend beyond the mitigation of risk factors, as physical fitness levels were found to be associated with lower cardiovascular and all‐cause mortality rates, even when independently adjusted for cardiovascular disease risk factors (Sandvik et al., 1993).

Recent emerging data has shown that physical fitness not only benefits parental hosts’ health, but also that of their offspring (Carter, Qi, De Cabo, & Pearson, 2013; Denham, 2018; Leite et al., 2017; McCullough et al., 2015; Yeshurun & Hannan, 2018). These findings have been observed in a variety of measures ranging from insulin sensitivity (Barres & Zierath, 2011) to anxiety profiles (Short et al., 2017). For example, maternal physical activity during pregnancy or paternal physical activity during the pre‐conception period was shown to improve offspring glucose tolerance (Carter et al., 2013; Stanford et al., 2018) with pre‐conception paternal exercise eliciting more favorable offspring body weight and C‐reactive protein profiles (McPherson, Owens, Fullston, & Lane, 2015).

**Figure 1:**
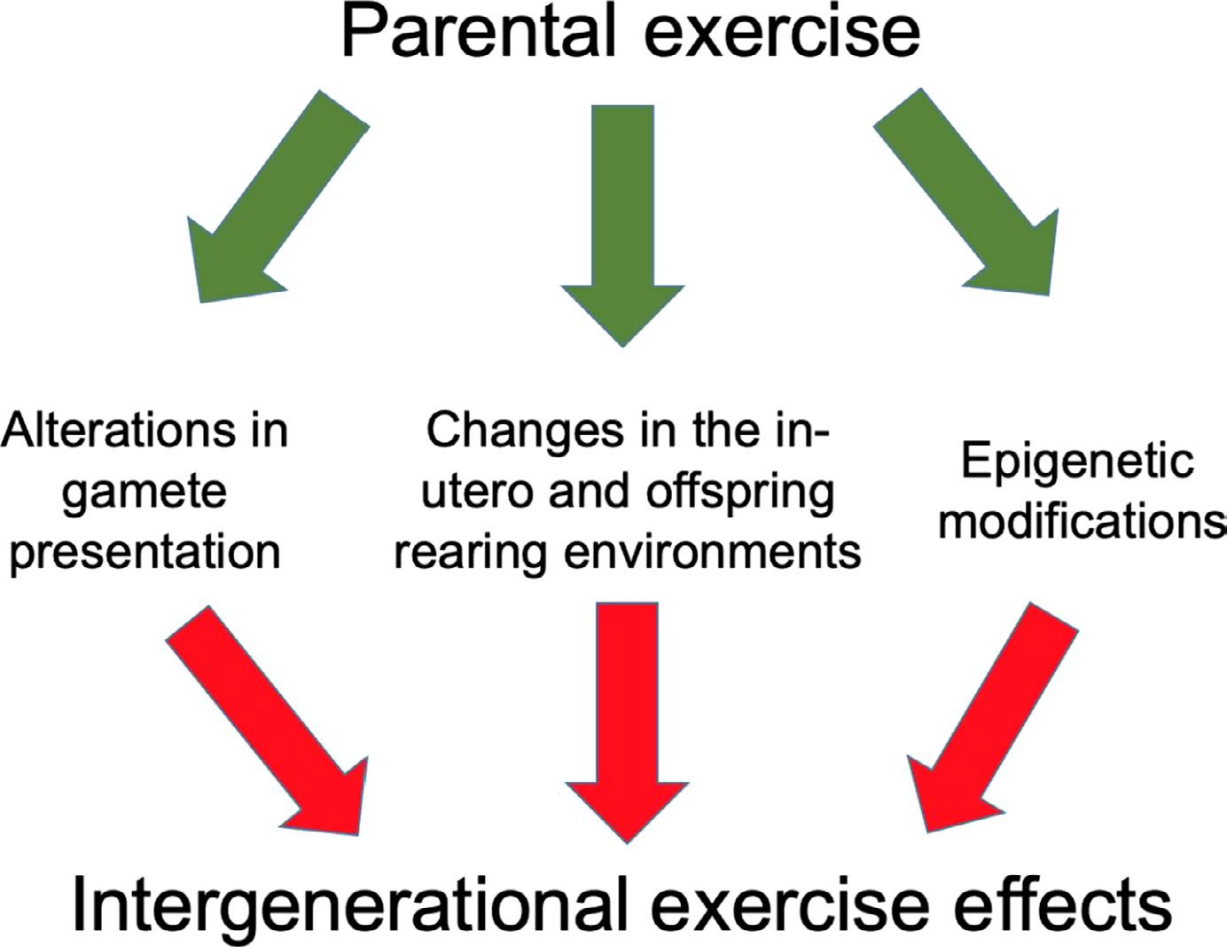
While it was previously thought an organism's epigenome was erased during gametogenesis and embryogenesis, it is now accepted that some epigenetic modifications are potentially heritable. Some mechanistic possibilities for the intergenerational effects of exercise are alterations in gamete presentation, changes in the in utero and offspring rearing environments, and epigenetic modifications

Several complex and interrelated mechanisms have been proposed for this intergenerational effect of exercise, including alterations in gamete presentation, changes in the in utero and offspring rearing environments, and epigenetic modifications (Figure 1). While it was previously thought an organism's epigenome was erased during gametogenesis and embryogenesis, it is now accepted that some epigenetic modifications are potentially heritable, able to be transmitted from parent to offspring (Portela & Esteller, 2010). The purpose of this brief systematic review was to expound upon epigenetic modulation of exercise and its intergenerational effects. Specifically, we will focus on the two most common epigenetics modifications, DNA methylation and histone modifications, in both humans and animal models (Portela & Esteller, 2010).

## 2 METHODS

### 2.1 Question

This review question was formulated using the evidence‐based model PICO: Population, Intervention, Comparison, Outcome. The population consisted of subjects engaged in reproduction, the intervention was exercise, the comparison was sedentary behavior, and the outcome was epigenetic modifications in offspring. Using these components, the question is as follows: Does paternal and/or maternal exercise status incur epigenetic modifications inherited by offspring?

### 2.2 Search strategy

Key words were identified for components of the PICO question (Table 1). The initial search was completed using Pubmed and yielded seven results. To garner a larger number of results, another search was completed in Pubmed using more broad key words (Table 2) which yielded 79 results. A second search also using these “wide net” key words was completed in Embase (Table 3).

**Table 1:** Identified key words for each component of the PICO question

Search Strategy	
Search Database	PubMed
Intervention	"Exercise" OR "Physical Activity" OR "Activity, Physical" OR "Acute Exercise" OR "Chronic Exercise" OR "Aerobic Exercise" OR "Exercise Training" OR "Isometric Exercise" OR "Animal Physical Conditioning" OR "Running" OR "Swimming"
Population	"Placenta" OR "Sperm" OR "Reproduction" OR "Paternal" OR "Maternal, Pregnancy" OR "Inheritance Patterns" OR "Genetic Phenomena"
Outcome	“Epigenesis, Genetic” OR “Epigenomics” OR “Process, Epigenetic” OR “DNA Methylations” OR “Methylation, DNA” OR “Acetyltransferases, Histone” OR “Acetylase, Histone” OR “Deacetylases, Histone” OR “Histone Deacetylase”
Search Result	*N* = 7

**Table 2:** Key words used in a systematic search of Pubmed literature

Search strategy	
Search Database	PubMed
Intervention	"Exercise" OR "Physical Activity"
Population	"Sperm" OR "Placenta" OR "Paternal" OR "Maternal" OR "Pregnancy"
Outcome	“Epigenetic”
Search Result	*N* = 79

**Table 3:** Key words used in a systematic search of Embase literature

Search strategy	
Search Database	Embase
Intervention	"Exercise"
Population	"Sperm" “Placenta” “Paternal Exposure” “Paternal Inheritance” “Paternal Behavior” “Maternal” “Offspring”
Outcome	“Epigenetic”
Search Result	*N* = 26

### 2.3 Inclusion and exclusion criteria

**Figure 2:**
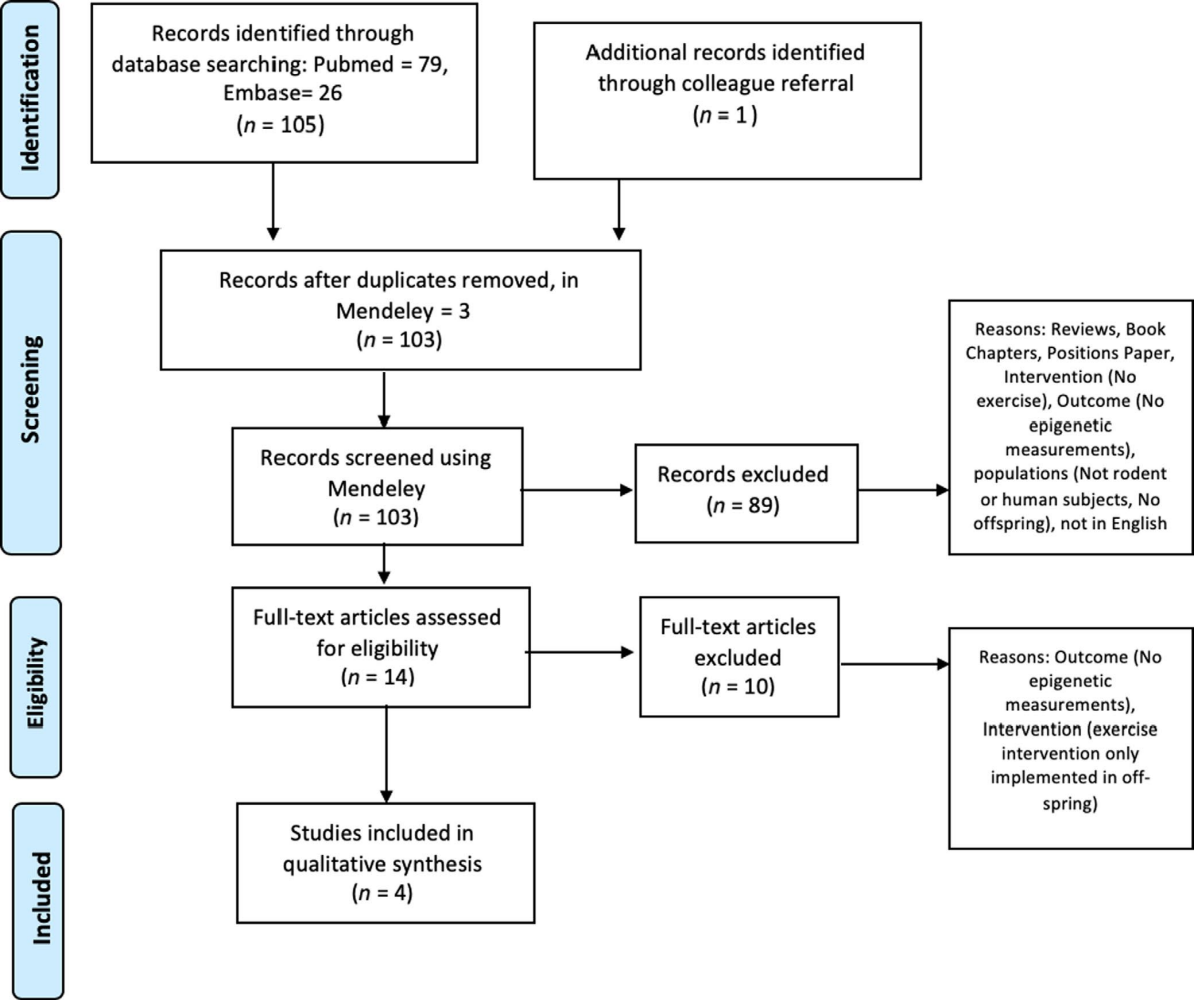
PRISMA diagram of flow of information through systematic review inclusion and exclusion criteria

Articles had to include original research. Reviews, book chapter, or position papers were excluded. Articles not in English were excluded. Articles were limited to the years 2008–2018. Study subjects had to be human or rodent models. Exercise had to be an intervention used in the maternal and/or paternal generation. Epigenetic mechanisms had to be measured in the offspring. A PRISMA diagram outlines these inclusion and exclusion criteria (Figure 2). A total of four articles met inclusion criteria.

## 3 RESULTS

Four studies met the inclusion criteria of original research available in English completed between 2008 and 2018 examining epigenetic outcomes of an exercise intervention in human or rodent offspring (Table 4). 

**Table 4:** Characteristics of the four studies that met the following experimental design inclusion criteria: founder exercise intervention and epigenetic modifications measured in offspring

Study	Population	Diet	Exercise Intervention	Methylation Levels	mRNA or Protein Expression	Functional Measurements
McCullough et al. (2015)	Female Humans	Standard	Prenatal physical activity levels assessed using the Pregnancy Physical Activity Questionnaire tool for 24 weeks	DNA methylation of differentially methylated regions (DMR) regulating four known imprinted genes with roles in fetal development (*H19, MEG3, SGCE/PEG10,* and *PLAGL1*) Association with prenatal physical activity (PA) after adjusting for race/ethnicity, gestational age at delivery, folic acid intake, maternal smoking	None measured	Association of prenatal PA with birth weight after adjusting for race/ethnicity and preterm birth
Laker et al. (2014)	Female C57BL/6 Mice	Two diet groups: high‐fat diet (HFD) versus control diet (CD) Length: 6 weeks before and throughout pregnancy	HFD group subdivided: sedentary versus access to a running wheel. Length: 6 weeks before and throughout pregnancy	Neonatal and 12‐month‐old offspring skeletal muscle Methylation of *Peroxisome proliferator‐activated‐receptor gamma coactivator‐alpha* (*Pgc−1 alpha*) at CpG site −260	Neonatal and 12‐month‐old offspring skeletal muscle mRNA levels	Glucose tolerance tests in 6, 9, and 12‐month‐old offspring
Xu et al. ( 2017)	Female Kunming Mice	Standard	Two activity groups: sedentary versus treadmill training Treadmill training occurred 5 days/week and 60 min/day Length: 4 weeks prior to superovulation and oocyte collection for in vitro fertilization (IVF)	Embryos (blastocyst stage) produced via IVF Methylation levels of CpG sites on imprinting control regions of maternal and paternalimprinted genes Global DNA methylation Histone methylation	mRNA levels of maternal or paternal imprinted genes	IVF‐derived embryos (blastocyst stage) H_2_O_2_, ATP, and Δψm measurements
Mega et al. (2018)	Male Wistar Rats	Standard	Two activity groups: sedentary versus treadmill training Treadmill training occurred for 20 min/day and 5 consecutive days/week Length: 8 weeks prior to mating	54‐day‐old male offspring Global DNA‐methylation of hippocampal tissue	54‐day‐old offspring BDNF protein quantification of hippocampal and cortex tissue	Male offspring at 1, 21, and 54 days old Body weight and growth Physical activity participation and performance Spatial memory and learning performance Adrenal gland, gonadal fat, and leg muscle weights

### 3.1 McCullough et al. (2015)

The first included study examined human female subjects’ physical activity levels with the Pregnancy Physical Activity Questionnaire tool for 24 weeks in the prenatal period. DNA methylation of previously identified differentially methylated regions (DMR) regulating imprinted genes were measured in human cord‐blood. Total maternal prenatal non‐sedentary time was associated with decreased DNA methylation of *Pleomorphic Adenoma Gene‐Like 1 (PLAGL1)* DMR (*P trend = .0136)* after adjusting for race/ethnicity, gestational age at delivery, folic acid intake, and maternal smoking*.* They also found an inverse relationship between moderate & intense physical activity and birth weight (BW). Conversely, light physical activity was positively associated with BW, but this relationship was attenuated with increasing amounts of light physical activity performed. 

### 3.2 Laker et al. (2014)

The second included study used a rodent model (C57BL/6 mice). Female mice were divided into a control diet (CD) group or a high‐fat diet (HFD) group. The high‐fat diet group was further subdivided into a sedentary group or a voluntary wheel access group. Both diet and exercise interventions were implemented 6 weeks before pregnancy and continued throughout. Offspring were either assessed during the neonatal period or at 12 months of age. DNA methylation of *Peroxisome proliferator‐activated‐receptor gamma coactivator‐alpha* (*Pgc‐1 alpha*) CpG site −260 was measured in skeletal muscle. High‐fat diet caused hypermethylation of *Pgc‐1 alpha* in neonates (*p* < .05), but this difference was completely abolished at 12 months of age in offspring of exercised‐founders. There were no differences in mRNA levels between groups in neonatal offspring, but by 12‐months of age both diet and exercise impacted mRNA levels. HFD‐exercise offspring had higher mRNA levels of Pgc‐1 alpha, Glut4*,* Cox4*,* and Cyt c compared to CD‐Sedentary and HFD‐sedentary offspring (*p* < .05). There was no difference in mRNA levels of Myh2a and Sod1 between groups. Longitudinal glucose tolerance tests (GTT) and insulin tolerance tests (ITT) were also conducted on offspring. Although there were no differences between exercise‐offspring and sedentary‐offspring at both 6 and 12 months, there was a significant difference at 9 months. HFD‐sedentary offspring possessed an impaired GTT, and HFD‐exercise offspring showed normal GTT (*p* < .05). 

### 3.3 Xu et al. (2017)

The third included study used female Kumming mice, divided into two groups (sedentary or treadmill training). The treadmill training occurred for 60 min/day, 5 days/week, and lasted 4 weeks prior to superovulation and oocyte collection for subsequent in vitro fertilization (IVF). Somatic nuclear cell transfer was also performed in separate experiments that are not discussed here due to lack of an intergenerational component. DNA‐methylation of CpG sites in select paternal or maternal‐imprinted genes, global DNA methylation, and histone methylation were measured in IVF‐derived embryos (blastocyst stage). Exercise‐embryos (EE) showed increased methylation of maternally‐imprinted *Igf2,* paternally‐imprinted *Meg3* & *H19,* and the histone H3K4m2, compared to sedentary‐embryos (SE) (*p* < .05). EE had lower methylation, however, of paternally‐imprinted *Igf2r*, global DNA methylation, and histone H3K9m3 (*p* < .05). EE also exhibited lower levels of maternally imprinted Igf2 mRNA and paternally imprinted Meg3 & H19 mRNA (*p* < .05). Yet EE possessed higher levels of paternally imprinted Igf2r and Ampd3 (*p* < .05). H_2_O_2_ level (pixels/embryo), ATP levels (pmol/embryo), and mitochondrial membrane potential (Δψm) were also assessed in offspring. Exercise‐embryos (EE) showed lower H_2_O_2_ levels yet higher ATP levels and mitochondrial membrane potentials (*p* < .05). 

### 3.4 Mega et al. (2018)

The fourth included study assessed male Wistar rats that were divided into sedentary or treadmill training groups. The treadmill training occurred 20 min/day, 5 consecutive days/week, and was implemented for 8 weeks prior to mating. Exercise‐offspring exhibited lower percent global DNA‐methylation in hippocampal tissue compared to sedentary offspring (*p* = .019). There were no differences in offspring hippocampal and cortex tissue quantified brain‐derived neurotrophic factor (BDNF) protein. There were also no differences in many of the functional outcomes assessed, including body weight, growth, physical activity participation or performance, spatial memory or learning performance, adrenal gland weight, or leg muscle weight. The exception was that exercise‐offspring had lower relative gonadal fat weight compared to sedentary‐offspring (*p* = .026).

## 4 DISCUSSION

To the best of our knowledge, this is the first review to systematically survey the literature for evidence of intergenerational exercise‐derived epigenetic modifications. While the number of studies is few, the literature shows altered methylation patterns, mRNA levels, and functional outcomes in offspring of exercised founders. Both global DNA methylation changes and promoter‐region methylation of specific genes differed in exercise‐offspring. Exercise was also found to attenuate or reverse some of the high‐fat diet associated methylation patterns. Many of the genes found to be differentially methylated are implicated in metabolic functions, such as regulation of oxidative metabolism and glucose transportation, which have obvious significance for offspring. In addition to metabolism, exercise has also been shown to impact cognitive development, as hippocampal DNA methylation was found to be lower in exercise‐offspring (Mega et al., 2018). Hence, epigenetic intergenerational outcomes appear to be consistent with epigenetic alterations in hosts undergoing exercise.

Both acute and chronic exercise interventions have been shown to induce a number of epigenetic modifications within the exercise host. In human skeletal muscle, decreased whole genome methylation and decreased methylation of promoter regions of key metabolic genes (i.e., *Pgc‐1 alpha*, *PDK4*, and *PPAR delta*) was observed after completing a single‐session of cycling at 80% of VO_2peak_ (Barres et al., 2012). Similarly, three months of regular single‐knee extension exercise showed genome‐wide DNA methylation alterations that were not observed in the untrained leg (Lindholm et al., 2014). Global‐DNA methylation changes were also reported in skeletal muscle following 6‐months of moderate intensity cycling and aerobic classes (Nitert et al., 2012). Twelve weeks of moderate‐intensity training was also shown to reduce global DNA methylation in peripheral mononuclear cells in elderly human subjects (Dimauro et al., 2016). In a study of 1,016 individuals over the age of 70, self‐reported physical activity levels were correlated with global DNA methylation even after adjusting for other contributing factors such as body mass index (BMI) or smoking (Luttropp, Nordfors, Ekstrom, & Lind, 2013).

McCullough et al. examined human subjects measured cord‐blood methylation in four previously identified DMRs associated with body weight and only one (*PLAGL1*) was found to have a significant association with prenatal physical activity levels (McCullough et al., 2015). Still, *PLAG1*, an important regulator of the cell cycle & apoptosis (Vega‐Benedetti et al., 2017), was found to have downstream effects on *Glut4, PPAR delta*, *PACAP1‐R*, and *Rasgrf1*, all which transcribe proteins involved in carbohydrate metabolism (Vega‐Benedetti et al., 2017), and linked with control of fetal growth and the pathogenesis of transient neonatal diabetes mellitus as well as certain types of cancer (Brøns et al., 2010; Hoffmann & Spengler, 2012; Vega‐Benedetti et al., 2017). McCullough et al. (2015) did find an inverse association between moderate and heavy prenatal physical activity and infant birth weight. While there are probably many contributing factors to this relationship, the lower methylation of *PLAGL1* associated with physical activity may have beneficial effects on fetal growth.

Studies in rodent models have also shown host epigenetic changes in response to exercise. An exhaustive rotarod session in mice was shown to shift methylation patterns of histones that contributed to upregulation of an exercise‐associated isoform of *PGC‐1 alpha*, a protein important for mitochondrial biogenesis (Lochmann, Thomas, Bennett, & Taylor, 2015). Treadmill training in mice was also shown to result in 200 genes with altered DNA methylation that negatively correlated with gene expression, indicating DNA methylation may play a role in skeletal muscle adaptation to exercise (Kanzleiter et al., (2015)). *AMPK alpha‐2*, an important nutrient‐sensing energy regulator, was hypermethylated after short‐term treadmill training in mice and this was accompanied by a decrease in AMPK alpha‐2 mRNA and protein expression (King‐Himmelreich et al., 2016). This hypermethylation was not present after a longer‐term intervention, suggesting it might have a transient role in adaptation to stimulus and highlighting the impact exercise duration, intensity, and length might have on epigenetic changes (King‐Himmelreich et al., 2016).

Changes in methylation levels might be expected to be accompanied by a shift in mRNA levels, as methylation makes the genome more or less available for transcription. mRNA levels both increased and decreased in exercise‐offspring depending on which transcripts were surveyed. While there was a lack of consensus across studies of which mRNAs transcripts were surveyed many have been found to have metabolic roles. Exercise increased levels of mRNA transcripts (even in the context of a high‐fat diet challenge) that could enhance offspring oxidative metabolism and regulation of growth. Notably, exercise increased levels mRNA transcript levels of *Pgc‐1 alpha* (Laker et al., 2014), a transcriptional coactivator which has been referenced as a “master regulator of mitochondrial function (Barres & Zierath, 2011)” and was more methylated in low‐birth weight infants (Brøns et al., 2010). Moreover with respect to phenotype, exercised‐offspring exhibited molecular‐level increases in ATP and mitochondrial membrane potential, both of which could help enhance metabolic function (Xu et al., 2017). While offspring of founders exposed to a high fat diet showed impaired glucose tolerance, exercise was able to rescue this impairment (Laker et al., 2014). When functional outcomes measured included cognitive changes there was no difference found between offspring of exercise‐founders versus sedentary‐founders (Mega et al., 2018). And while there were no differences found in rodent‐model offspring body weight, there was an inverse relationship found between moderate & intense prenatal‐PA and birth weight when studying human participants (McCullough et al., 2015).

A number of studies have also reported epigenetic modifications in exercised‐founder sperm (Benito et al., 2018; Ingerslev et al., 2018; Murashov et al., 2015; Stanford et al., 2018). A high‐fat diet caused changes in founder sperm content including altered methylation levels, microRNA (miRNA) content, and transfer ribonucleic acid fragments (tRFs) (Murashov et al., 2015; Stanford et al., 2018). Exercise interventions ameliorated these changes (Murashov et al., 2015; Stanford et al., 2018). Several of the methylated regions and miRNAs studied have metabolic roles such in glucose and insulin signaling. Even in the absence of diet interventions, exercise modified DMRs and piwi‐interacting RNAs (pi‐RNA) (Ingerslev et al., 2018; Stanford et al., 2018).

Given that exercise impacts sperm, it is reasonable to hypothesize that also has the potential to induce intergenerational effects. Offspring of high‐fat diet founders experienced metabolic dysfunction that was improved when founders were exercised (Stanford et al., 2018). Murashov et al. (2015) challenged their offspring (in addition to founders) with a high‐fat diet. When this F1 generation intervention was added, male offspring of male exercise‐founders showed more metabolic dysfunction than male offspring of male sedentary‐founders, while there was no impact in female offspring. This result highlights the complexity of metabolic function and the large impact of variables such as gender, which warrants further exploration. Benito et al. (2018) found offspring of exercise‐founders displayed increased hippocampal long‐term potentiation, a marker of synaptic function. Assessing epigenetic modifications in both sperm content and offspring could bolster connecting them to observed phenotypic changes.

Several limitations exist in both analyzing the current literature and in the field of intergenerational‐epigenetics in general. There are a clear lack of uniform methods across included studies. The length of exercise intervention ranged from 3 to 24 weeks, a variable that could have a large impact on resulting conclusions. There were also discrepancies between whether exercise was used as a stand‐alone intervention or was implemented along with changes to diet and environment. Measured outcomes were inconsistent between studies, making it difficult to compare results and assess studies for reproducibility. All of the studies except two used rodent models. Of the two that did not, one measured outcome in sperm (not offspring) and the other measured outcomes in offspring cord‐blood. Obviously, using human subjects makes it difficult to tightly control for the environment and other confounding variables. This is especially relevant in the field of intergenerational epigenetics, as it is extremely difficult to show observed modifications in offspring that are due solely to epigenetic modifications. When using female subjects, fetal developmental programming in utero could be responsible for many intergenerational effects. It would be prudent in future studies to examine offspring to the F3 or later generations (Horsthemke, 2018).

Much more research is needed to elucidate the potential for exercise to induce epigenetic modifications that have intergenerational effects. Standardization of exercise interventions employed, including length, mode, intensity, and sex, would help shed light on this important topic. Similarly, more uniformity in outcome measurements such as tissue sampled, global versus gene‐specific surveillance, and offspring sex could improve ability to compare results between studies. Most importantly, rigorous methods are needed to ensure the greatest likelihood of observed outcomes being attributable to epigenetic modifications. Suggested methods in translational models include using in‐bred strains, strictly controlling the environment, studying multiple generations of offspring, and studying germ cells as well as offspring (Horsthemke, 2018). Regardless of whether the intergenerational impacts of exercise are due to epigenetic modifications or fetal developmental programming, it could serve as a non‐invasive, low‐cost intervention during preconception or prenatal periods to help enhance various functional outcomes in offspring. Previous studies have found physical activity interventions during pregnancy are feasible and well tolerated by participants (Callaway et al., 2010; Hunter, 2017). While further studies are needed, these early data suggest exercise can impact intergenerational health through epigenetic modifications.

## CONFLICTS OF INTEREST

None of the authors have any conflicts of interest to declare, financial or otherwise.

## References

[phy214287-bib-0001] Barres, R. , Yan, J. , Egan, B. , Treebak, J. T. , Rasmussen, M. , Fritz, T. , … Zierath, J. R. (2012). Acute exercise remodels promoter methylation in human skeletal muscle. Cell Metabolism, 15, 405–411. 10.1016/j.cmet.2012.01.001 22405075

[phy214287-bib-0002] Barres, R. , & Zierath, J. R. (2011). DNA methylation in metabolic disorders. American Journal of Clinical Nutrition, 93, 897–900 (Suppl.) 10.3945/ajcn.110.001933 21289222

[phy214287-bib-0003] Benito, E. , Kerimoglu, C. , Ramachandran, B. , Pena‐Centeno, T. , Jain, G. , Stilling, R. M. , … Dean, C. (2018). RNA‐dependent intergenerational inheritance of enhanced synaptic plasticity after environmental enrichment. Cell Reports, 23(2), 546–554. 10.1016/j.celrep.2018.03.059 29642011 PMC5912949

[phy214287-bib-0004] Brøns, C. , Jacobsen, S. , Nilsson, E. , Ronn, T. , Jensen, C. B. , Storgaard, H. , … Vaag, A. (2010). Deoxyribonucleic acid methylation and gene expression of PPARGC1A in human muscle is influenced by high‐fat overfeeding in a birth‐weight‐dependent manner. The Journal of Clinical Endocrinology & Metabolism, 95(6), 3048–3056. 20410232 10.1210/jc.2009-2413

[phy214287-bib-0005] Callaway, L. K. , Colditz, P. B. , Byrne, N. M. , Lingwood, B. E. , Rowlands, I. J. , Foxcroft, K. … Bambino Group (2010). Prevention of gestational diabetes: Feasibility issues for an exercise intervention in obese pregnant women. Diabetes Care, 33(7), 1457–1459. 10.2337/dc09-2336 20357374 PMC2890340

[phy214287-bib-0006] Carter, L. G. , Qi, N. R. , De Cabo, R. , & Pearson, K. J. (2013). Maternal exercise improves insulin sensitivity in mature rat offspring. Medicine and Science in Sports and Exercise, 45(5), 832 10.1249/MSS.0b013e31827de953 23247711 PMC3617068

[phy214287-bib-0007] Denham, J. (2018). Exercise and epigenetic inheritance of disease risk. Acta Physiologica, 222(1), e12881 10.1111/apha.12881 28371392

[phy214287-bib-0008] Dimauro, I. , Scalabrin, M. , Fantini, C. , Grazioli, E. , Beltran Valls, M. R. , Mercatelli, N. , … Caporossi, D. (2016). Resistance training and redox homeostasis: Correlation with age‐associated genomic changes. Redox Biology, 10, 34–44. 10.1016/j.redox.2016.09.008 27687219 PMC5040637

[phy214287-bib-0009] Hoffmann, A. , & Spengler, D. (2012). Transient neonatal diabetes mellitus gene Zac1 impairs insulin secretion in mice through Rasgrf1. Molecular and Cellular Biology, 32, 2549–2560. 10.1128/MCB.06637-11 22547676 PMC3434484

[phy214287-bib-0010] Horsthemke, B. (2018). A critical view on transgenerational epigenetic inheritance in humans. Nature Communications, 9(1), 2973 10.1038/s41467-018-05445-5 PMC6065375 30061690

[phy214287-bib-0011] Hunter, B. (2017). Eat well keep active: Qualitative findings from a feasibility and acceptability study of a brief midwife led intervention to facilitate healthful dietary and physical activity behaviours in pregnant women. Midwifery, 49, 117–123. 27964858 10.1016/j.midw.2016.12.002

[phy214287-bib-0012] Ingerslev, L. R. , Donkin, I. , Fabre, O. , Versteyhe, S. , Mechta, M. , Pattamaprapanont, P. , … Barrès, R. (2018). Endurance training remodels sperm‐borne small RNA expression and methylation at neurological gene hotspots. Clinical Epigenetics, 10(1), 12 10.1186/s13148-018-0446-7 29416570 PMC5785820

[phy214287-bib-0014] Kanzleiter, T. , Jähnert, M. , Schulze, G. , Selbig, J. , Hallahan, N. , Schwenk, R. W. , & Schürmann, A. (2015). Exercise training alters DNA methylation patterns in genes related to muscle growth and differentiation in mice. American Journal of Physiology‐Endocrinology and Metabolism, 308(10), E912–E920. 10.1152/ajpendo.00289.2014 25805191

[phy214287-bib-0015] King‐Himmelreich, T. S. , Schramm, S. , Wolters, M. C. , Schmetzer, J. , Möser, C. V. , Knothe, C. , … Niederberger, E. (2016). The impact of endurance exercise on global and AMPK gene‐specific DNA methylation. Biochemical and Biophysical Research Communications, 474(2), 284–290. 10.1016/j.bbrc.2016.04.078 27103439

[phy214287-bib-0016] Laker, R. C. , Lillard, T. S. , Okutsu, M. , Zhang, M. , Hoehn, K. L. , Connelly, J. J. , & Yan, Z. (2014). Exercise Prevents Maternal high‐fat diet‐induced Hypermethylation of the Pgc‐1α Gene and age‐dependent Metabolic Dysfunction in the Offspring. Diabetes, DB_131614. 10.2337/db13-1614 PMC5860829 24430439

[phy214287-bib-0017] Lee, D. C. , Sui, X. , Church, T. S. , Lavie, C. J. , Jackson, A. S. , & Blair, S. N. (2012). Changes in fitness and fatness on the development of cardiovascular disease risk factors: Hypertension, metabolic syndrome, and hypercholesterolemia. Journal of the American College of Cardiology, 59(7), 665–672. 10.1016/j.jacc.2011.11.013 22322083 PMC3293498

[phy214287-bib-0018] Leite, C. F. , do Nascimento, S. L. , Helmo, F. R. , dos Reis Monteiro, M. L. G. , dos Reis, M. A. , & Corrêa, R. R. M. (2017). An overview of maternal and fetal short and long‐term impact of physical activity during pregnancy. Archives of Gynecology and Obstetrics, 295(2), 273–283. 10.1007/s00404-016-4204-9 27761731

[phy214287-bib-0019] Lindholm, M. E. , Marabita, F. , Gomez‐Cabrero, D. , Rundqvist, H. , Ekstrom, T. J. , Tegner, J. , & Sundberg, C. J. (2014). An integrative analysis reveals coordinated reprogramming of the epigenome and the transcriptome in human skeletal muscle after training. Epigenetics, 9, 1557–1569. 10.4161/15592294.2014.982445 25484259 PMC4622000

[phy214287-bib-0020] Liu, X. , Tong, Z. , Chen, K. , Hu, X. , Jin, H. , & Hou, M. (2018). The Role of miRNA‐132 against apoptosis and oxidative stress in heart failure. Biomed Research International, 2018 10.1155/2018/3452748 PMC5845498 29682535

[phy214287-bib-0021] Lochmann, T. L. , Thomas, R. R. , Bennett, J. P. Jr , & Taylor, S. M. (2015). Epigenetic modifications of the PGC‐1α promoter during exercise induced expression in mice. PLoS ONE, 10(6), e0129647 10.1371/journal.pone.0129647 26053857 PMC4460005

[phy214287-bib-0022] Luttropp, K. , Nordfors, L. , Ekstrom, T. J. , & Lind, L. (2013). Physical activity is associated with decreased global DNA methylation in Swedish older individuals. Scandinavian Journal of Clinical and Laboratory Investigation, 73, 184–185. 10.3109/00365513.2012.743166 23171428

[phy214287-bib-0023] McCullough, L. E. , Mendez, M. A. , Miller, E. E. , Murtha, A. P. , Murphy, S. K. , & Hoyo, C. (2015). Associations between prenatal physical activity, birth weight, and DNA methylation at genomically imprinted domains in a multiethnic newborn cohort. Epigenetics, 10(7), 597–606. 10.1080/15592294.2015.1045181 25928716 PMC4622928

[phy214287-bib-0025] McPherson, N. O. , Owens, J. A. , Fullston, T. , & Lane, M. (2015). Preconception diet or exercise intervention in obese fathers normalizes sperm microRNA profile and metabolic syndrome in female offspring. American Journal of Physiology‐Endocrinology and Metabolism, 308(9), E805–E821. 10.1152/ajpendo.00013.2015 25690453

[phy214287-bib-0026] Mega, F. , de Meireles, A. L. F. , Piazza, F. V. , Spindler, C. , Segabinazi, E. , dos Santos Salvalaggio, G. , … Marcuzzo, S. (2018). Paternal physical exercise demethylates the hippocampal DNA of male pups without modifying the cognitive and physical development. Behavioural Brain Research, 348, 1–8. 10.1016/j.bbr.2018.03.040 29614250

[phy214287-bib-0027] Murashov, A. K. , Pak, E. S. , Koury, M. , Ajmera, A. , Jeyakumar, M. , Parker, M. , … Neufer, P. D. (2015). Paternal long‐term exercise programs offspring for low energy expenditure and increased risk for obesity in mice. The FASEB Journal, 30(2), 775–784. 10.1096/fj.15-274274 26506979 PMC4714554

[phy214287-bib-0028] Nitert, M. D. , Dayeh, T. , Volkov, P. , Elgzyri, T. , Hall, E. , Nilsson, E. , … Ling, C. (2012). Impact of an exercise intervention on DNA methylation in skeletal muscle from first‐degree relatives of patients with type 2 diabetes. Diabetes, 61, 3322–3332. 10.2337/db11-1653 23028138 PMC3501844

[phy214287-bib-0029] Nocon, M. , Hiemann, T. , Müller‐Riemenschneider, F. , Thalau, F. , Roll, S. , & Willich, S. N. (2008). Association of physical activity with all‐cause and cardiovascular mortality: A systematic review and meta‐analysis. European Journal of Cardiovascular Prevention & Rehabilitation, 15(3), 239–246. 10.1097/HJR.0b013e3282f55e09 18525377

[phy214287-bib-0030] Portela, A. , & Esteller, M. (2010). Epigenetic modifications and human disease. Nature Biotechnology, 28, 1057–1068. 10.1038/nbt.1685 20944598

[phy214287-bib-0031] Sandvik, L. , Erikssen, J. , Thaulow, E. , Erikssen, G. , Mundal, R. , & Rodahl, K. (1993). Physical fitness as a predictor of mortality among healthy, middle‐aged Norwegian men. New England Journal of Medicine, 328(8), 533–537. 10.1056/NEJM199302253280803 8426620

[phy214287-bib-0032] Short, A. K. , Yeshurun, S. , Powell, R. , Perreau, V. M. , Fox, A. , Kim, J. H. , … Hannan, A. J. (2017). Exercise alters mouse sperm small noncoding RNAs and induces a transgenerational modification of male offspring conditioned fear and anxiety. Translational Psychiatry, 7, e1114–e1122. 10.1038/tp.2017.82 28463242 PMC5534950

[phy214287-bib-0033] Stanford, K. I. , Rasmussen, M. , Baer, L. A. , Lehnig, A. C. , Rowland, L. A. , White, J. D. , … Rando, O. J. (2018). Paternal exercise improves glucose metabolism in adult offspring. Diabetes, 67(12), 2530–2540. 10.2337/db18-0667 30344184 PMC6245224

[phy214287-bib-0034] Vega‐Benedetti, A. F. , Saucedo, C. , Zavattari, P. , Vanni, R. , Zugaza, J. L. , & Parada, L. A. (2017). PLAGL1: An important player in diverse pathological processes. Journal of Applied Genetics, 58(1), 71–78. 10.1007/s13353-016-0355-4 27311313

[phy214287-bib-0035] Xu, W. H. , Wu, H. , Xia, W. L. , Lan, H. , Wang, Y. , Zhang, Y. , & Hua, S. (2017). Physical exercise before pregnancy helps the development of mouse embryos produced in vitro. Mitochondrion, 34, 36–42. 10.1016/j.mito.2016.12.004 28017685

[phy214287-bib-0036] Yeshurun, S. , & Hannan, A. J. (2018). Transgenerational epigenetic influences of paternal environmental exposures on brain function and predisposition to psychiatric disorders. Molecular Psychiatry, 1. 10.1038/s41380-018-0039-z 29520039

